# Exploring patient perspectives on the secondary use of their personal health information: an interview study

**DOI:** 10.1186/s12911-023-02143-1

**Published:** 2023-04-11

**Authors:** Rosie Dobson, Helen Wihongi, Robyn Whittaker

**Affiliations:** 1 School of Population Health, University of Auckland, Auckland, New Zealand; 2 Te Whatu Ora Waitematā, Auckland, New Zealand; 3 Te Whatu Ora Te Toka Tumai, Auckland, New Zealand

**Keywords:** Health information, Data sharing, Consumer perspectives

## Abstract

**Background:**

The increased digitalisation of health records has resulted in increased opportunities for the secondary use of health information for advancing healthcare. Understanding how patients want their health information used is vital to ensure health services use it in an appropriate and patient-informed manner. The aim of this study was to explore patient perceptions of the use of their health information beyond their immediate care.

**Methods:**

Semi-structured in-depth interviews were conducted with current users of health services in Aotearoa New Zealand. Different scenarios formed the basis of the discussions in the interviews covering different types of information use (current practice, artificial intelligence and machine learning, clinical calculators, research, registries, and public health surveillance). Transcripts were analysed using thematic analysis.

**Results:**

Twelve interviews were conducted with individual’s representative of key ethnicity groups and rural/urban populations, and at the time of recruitment, had been accessing a diverse range of health services. Participants ranged from high users of health care (e.g., weekly dialysis) through to low users (e.g., one-off presentation to the emergency department). Four interrelated overarching themes were identified from the transcripts describing the main issues for participants: helping others, sharing of data is important, trust, and respect.

**Conclusions:**

People currently engaging with health services are supportive of their health information being used to help others, advance science, and contribute to the greater good but their support is conditional. People need to be able to trust the health service to protect, care for, and respect their health information and ensure no harm comes from its use. This study has identified key considerations for services and researchers to reflect on when using patient health information for secondary purposes to ensure they use it in a patient-informed way.

**Trial registration:**

NA.

**Supplementary Information:**

The online version contains supplementary material available at 10.1186/s12911-023-02143-1.

## Background

Secondary use of health information is fundamental for improving health services and has the potential to not only progress health outcomes but support addressing health inequalities. Health information has informed clinical care for generations, but the increased digitalisation of health records and processes has increased the opportunities for improving processes (e.g., automated clinical calculators and decision support prompts and pathways) and for advancing new opportunities (e.g., artificial intelligence (AI)). Understanding how patients want their own health information accessed and used is vital to ensure health services use it in an appropriate and patient-informed manner.

Although previous research has explored public and patient perspectives on this, the complexities around new innovations as well as the changing landscape mean perceptions are fluid. For example, the use of health information in the public health response to COVID-19 provided public exposure to the use of aggregated health data on a scale arguably not seen before. It has been found that patients were more comfortable with the sharing of health information with health-related (non-commercial) organisations during the pandemic than prior to the pandemic,[1] although there are conflicting reports around levels of comfort with the sharing of health information for purposes related to COVID-19.[1–4].

Generally, evidence to date has shown public support for secondary uses of health information on the condition that its use is for public benefit.[5–9] Although the need for individual consent for secondary use has been found to not always be necessary, concerns have been identified around privacy, security, misuse of data, and commercial use.[5, 6, 10, 11] Research has highlighted the need to improve patient confidence in their health services’ ability to protect their health information with greater transparency around processes and governance.[12–14].

In Aotearoa New Zealand (NZ) the increased digitisation of health records has been coupled with a growing interest in the use of health information for new innovations to improve existing processes and procedures, and for research. In these contexts, there is often a need for involvement of organisations or individuals outside a patient’s direct clinical team and it is not always possible to obtain individual informed consent for its use. Previous work in NZ has shown that both patients and the general public are largely comfortable with the secondary use of their de-identified health information.[6, 15] A survey of 1,377 current patients found that over 80% were comfortable with their health information being used across a range of scenarios. Comfort with the use of individual health information was related to assurances that its use was for public good, data was stored securely, individual privacy was maintained, and there was communication on how it was used.[6] The same survey administered to a general public cohort (rather than current patients of a health service; n = 2,572) found similar results with levels of comfort also being dependent on data not being shared outside the health system or used for commercial gain.[15] Both surveys highlighted that there was a current lack of communication and transparency around health information use.

This study was designed to build on this previous work by investigating more in-depth perspectives on the use of, and access to, individual healthcare information in people currently engaged with secondary care hospital services in NZ.

## Methods

The aim of this study was to explore patient perceptions of the use of their health information beyond their immediate care. For this observational study, semi-structured interviews were conducted with current users of services within a large secondary care health district in Auckland NZ (Te Whatu Ora Waitematā). Ethical approval for this study was obtained from the NZ Health and Disability Ethics Committee (20/NTA/2). Research approval from Waitematā District Health Board was obtained. This qualitative interview study uses a general inductive approach to understand patient preferences and perspectives,[16] and the reporting follows the Consolidated Criteria for Reporting Qualitative research (COREQ).[17].

### Context

This study was conducted in one of the 20 districts (formally known as District Health Boards) in the NZ public health system. Secondary care health services (inpatient and outpatient) are free within the public health system. Each health district is responsible for the protection and care of the information of patients who use their services.[18] At the time the study commenced the region was in a strict lockdown due to outbreak of the delta variant of COVID-19 but by the end of data collection restrictions had been eased.[19].

### Inclusion criteria

The specific inclusion criteria were (1) current user of Te Whatu Ora Waitematā inpatient and outpatient services, (2) 16 years or over, and (3) able to provide consent to participate. These inclusion criteria were the same as the previous study,[6] with the intention of the sample to be broadly representative of adult patients of the health services (both admitted to hospital and those attending clinics). By recruiting participants at the time of an encounter with a health service it was considered that they would have some understanding of the nature of the health information collected and the potential uses of such information.

### Procedures

Due to COVID-19 government imposed public health restrictions, in person recruitment within the health service was not possible and all study procedures had to be conducted remotely. Instead, clinicians were approached to ask if they could identify potential participants and get their permission to be contacted about the study. Potential participants were then contacted by phone by the researcher to discuss the study, answer questions and obtain verbal consent to participate.

Interviews were conducted by a female trained researcher (RD) who has extensive experience in interviewing. The interviewer had no relationship with or prior knowledge of the participants. Interviews were conducted over the phone or via zoom according to the participant’s preference and were able to be conducted over more than one sitting if preferred. A small voucher was provided to participants to acknowledge their time.

Recruitment continued until it was determined that a representative sample across demographic groups and health services had been obtained, we had reached data sufficiency (additional interviews were not adding new information), and the quality of the dialogue indicated a sufficient level of informational power.[20] Interviews were audio-recorded, transcribed by an independent transcriber and de-identified before analysis.

### Interview guide

The interviews were designed to be semi-structured. The participants were presented with 6 different scenarios and prompted to discuss their thoughts on the scenarios and any concerns or parts they may be uncomfortable with. Participants were also prompted to discuss whether their views were the same if it was their family/whānau health information and if the health information continued to be used after they had passed away. The interview guide can be found in Supplementary File 1 and short summaries of the six scenarios are below:

1. Current use of health information: Clinicians use a person’s health information to guide their current treatment and care. They share the information with other people who look after that person’s healthcare such as their general practitioner or clinicians at another hospital. Their de-identified information is used for statistics which are used to monitor the health service to ensure that they are running smoothly. For health information to be used in other ways, such as for research, typically the individual will have to provide consent for this and if they were not able to be contacted, their health information would not be used.
2. Machine learning (ML) and AI: The use of health information (e.g., mammogram images and results) for ML or to develop AI. By using the data of a large number of patients who have had a mammogram previously it is possible to develop computer programmes that can read mammograms of people who have breast screening making the future diagnosis of breast cancer more accurate, quicker, and cheaper than traditional methods. Within this scenario the following were also explored:
  Linking existing health information (e.g., past mammograms) with future health information (e.g., future diagnosis of breast cancer).
  External companies being involved in the development of the computer programmes and using the health information to create other computer programmes for other health providers (e.g., another hospital in another country).
  External companies selling the computer programme to make a profit.

3. Registries: The adding of de-identified health information to registries (e.g., a cardiac register if you have had a heart attack). Clinicians and researchers can then look at trends and outcomes for people in the registry to learn things like the types of people having heart attacks or the outcomes of certain treatments in certain types of people. Within this scenario the following was also explored:
  The re-identification of individuals from the registry and them being contacted by someone not from their medical/clinical team (e.g., a researcher from a university or a doctor from a different hospital) to be offered new treatments/services/research studies.

4. Clinical calculators: The use of de-identified health information from all relevant patients (including deceased patients) to create NZ specific calculators (e.g., a calculator for determining whether someone should get a kidney transplant). To be able to create NZ specific calculators they would need to be able to access the health information of all the people who had had the condition at the health service even if they had passed away. It would not be possible to consent each individual for the use of their data in this way.
5. Research: The use of deidentified data for research studies. To investigate recovery from hip replacement surgery, researchers might want to access the health information of everyone who underwent that surgery in a hospital in a particular year. This would be de-identified data but include details such as demographics, existing health conditions/diseases, specific test results, medications, allergies, and surgery outcomes. They may then try improving the service (e.g., changing the physiotherapy given post-surgery) and monitor any changes in the data to see whether it has been successful. This sharing of health information would be for the benefit of others rather than the participant. Within this scenario the following were also explored:
  Sharing the de-identified data with external researchers or clinicians from partner institutions (e.g., University of Auckland) to collaborate on the research or do the analysis.
  Sharing of the health information with institutions outside NZ (e.g., an Australian university) and therefore a non-New Zealander interpreting the data.

6. Public health: The sharing of health information in relation to COVID-19 surveillance. This involved the health service sharing identifiable information with other organisations within the wider health system (e.g., the Ministry of Health or the Regional Public Health Service) who would then make some of the de-identified information available to the public and media (e.g., genetic variant, infectious period, places visited while infectious).

Following discussion of the scenarios, participants were also asked questions related to (1) consent to use health information, including when and how consent should be obtained, and how often people should be asked to review/provide consent; (2) How, when and where patients should have access to their own hospital-held health information; and (3) How health services should communicate about the use of personal health information.

### Reflexive statement

RD holds a PhD and is a psychologist and senior research fellow, RW holds a PhD and is a professor and public health physician, and HW holds a PhD in psychology and is the Director of a Māori Health Research Department. All work across both academic and health service settings, including in the health district in which this study was carried out.

### Analysis

Transcripts were analysed using thematic analysis.[21, 22] The analysis process followed the phases outlined by Braun and Clarke (2021), initially, one team member (RD) became familiar with the interview transcripts noting initial ideas and themes and then undertook initial data coding across the whole dataset. Initial codes were then collated into potential themes by two team members (RD, RW) and cross-checking of the themes with coded extracts and the data set occurred. Ongoing analysis to refine the themes, including naming and defining the themes, was carried out by two team members (RD, RW). Themes’ names and definitions were also discussed with the wider research team and the Waitematā Artificial Intelligence Governance Group (members include representation of consumers/patients, clinical governance, data and digital governance, privacy, and security, legal, Māori and Pacific Island health, research, analytics, innovation and improvement, and expertise in AI/ML), and further feedback and clarifications were incorporated. A summary of the results, including the themes and their definitions, were shared with the participants and discussion/feedback welcomed. Although offered no participants requested a copy of their transcript.

## Results

There were 16 individuals referred to the study, with a total of 12 interviews conducted between October 2021 and February 2022. Of the remaining four people referred, three declined to participate and one was unable to be contacted. Interviews ranged in duration from 26 to 121 min (mean = 57 min). Participants ranged in age from 25 to 77 years and were representative of key ethnicity groups, rural/urban populations, and at the time of recruitment had been accessing a diverse range of health services. Participants ranged from high users of health care services (e.g., weekly dialysis) through to low users (e.g., one-off presentation to the emergency department).The demographic information of participants can be seen in Table 1.

**Table 1:** Demographic information of participants (n = 12)

	n	%
**Age group**		
≤ 34	2	17%
35–54	4	33%
55–74	4	33%
≥ 75	2	17%
**Age (Mean (SD), range)**	55.08 (15.86)	25–77
**Gender**		
Male	4	33%
Female	8	67%
**Ethnicity (prioritised, L1)**		
European	9	75%
Māori	1	8%
Pacific peoples	1	8%
Asian	1	8%
**Locality**		
Rural	3	25%
Urban	9	75%
**Encounter at time of recruitment**		
Renal services	3	25%
Physiotherapy outpatient services	2	17%
Emergency department	2	17%
Cardiology services	1	8%
Dental service	1	8%
Haematology service	1	8%
Mental health services	1	8%
Maternity services	1	8%

**Fig. 1:**
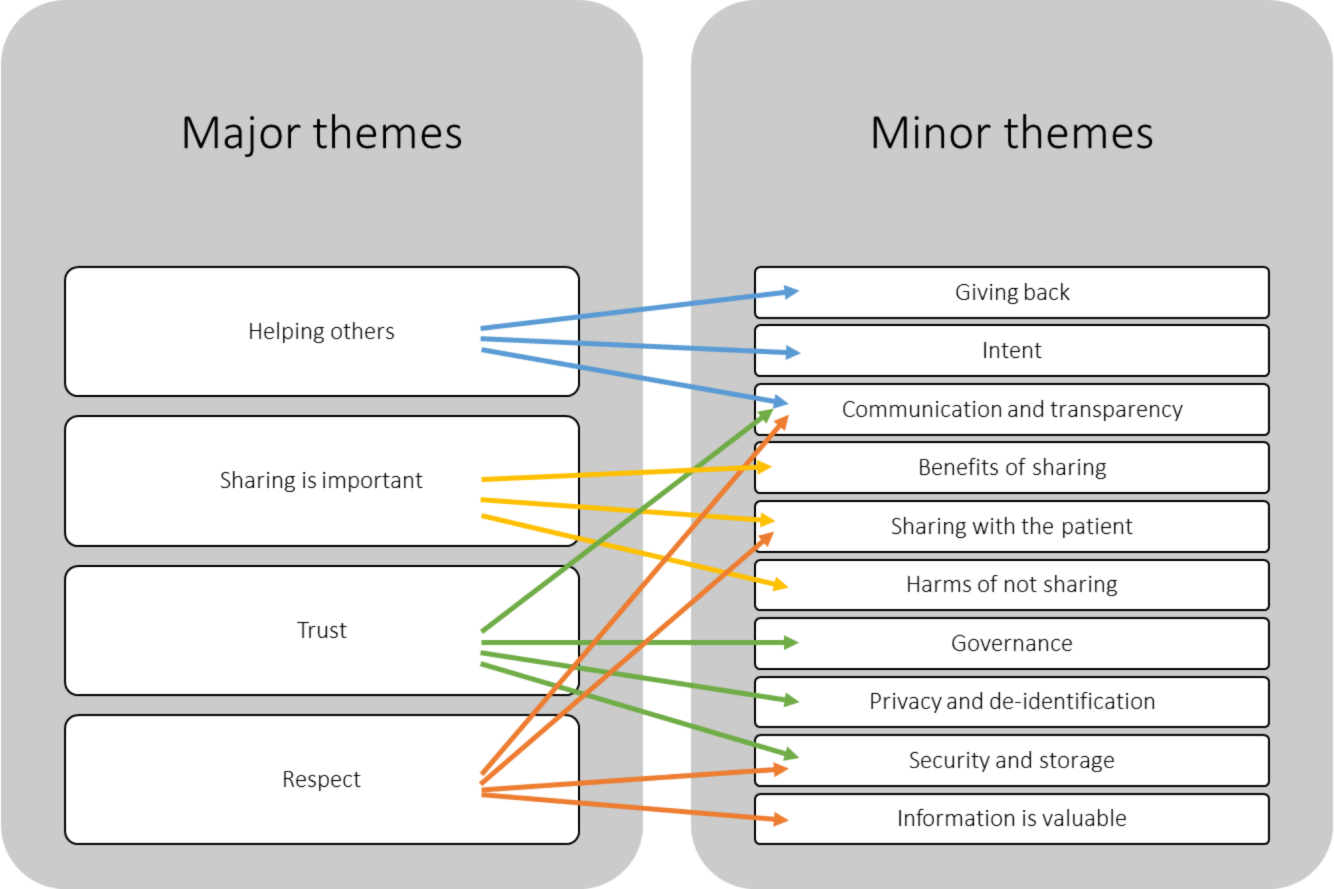
Coding tree of themes and subthemes

Four interrelated overarching themes were identified from the transcripts describing the main issues for participants: helping others, sharing of data is important, trust, and respect. Across the four themes a total of 10 sub-themes were identified. The relationships between the major and minor themes are displayed in Fig. 1. Each theme is discussed separately below, and quotes included to illustrate the themes.

### Theme 1: helping others

Across all scenarios participants described a desire for their health information to be used to help and benefit others.

*“I have personally no issue with it, because it’s for the greater good.” [Participant 12]*

*“If it saves people’s lives, I’m all for it.” [Participant 03]*

*“I don’t think it’s a bad idea, especially if it’s going to help our people – not only the generation that we are in now, but the next coming generation and the next one, because we’re moving forward, not backwards. So, I guess that will help us now, maybe – and the future.” [Participant 09]*

Their comfort with the use of their health information in the ways discussed in the scenarios was dependent on it benefiting others. In some cases, this benefit to others was seen as outweighing any personal discomfort with its use. For example, the disclosure of health information in relation to public health measures was described as something that people might be embarrassed by, but they felt that this discomfort was necessary as the potential benefit to the public outweighed the discomfort.

*“I’d have to just bite my tongue and let them get on with it. It’s important to other people.” [Participant 03]*

Beyond the immediate benefit of helping others, participants also described that the use of health information had secondary benefits which would also contribute to better outcomes for the wider community. For example, they described the use of data for ML and AI could result in more efficient and timely services therefore freeing up clinicians to care for their patients in other ways.

#### Giving back

Participants described an awareness that they had benefited from the secondary use of patient health information in the past. They were aware that others’ health information had been used to help develop the treatments and services they were using/receiving, and therefore the use of their health information would benefit others in the future by contributing to the development of new treatments and improving services. They saw their information being used as a way of giving back to the system they had benefited from.

*“I think that’s a good idea. You still are benefiting in a way, because the way you get treated is based on information from previous people, so you’re giving back. So, I think it would be again, a valid contribution to the community.” [Participant 10]*

*“I think it helps all families, because I think a lot of us have health issues that run on for generations, so I think that’s fine. I actually think it would help a lot.” [Participant 02]*

Participants saw the potential benefit of the use of their health information extending beyond their death. They understood the value of their health information for years to come. For example, with the clinical calculator’s scenario, participants appeared to understand the value of complete datasets and that it was important that the data of those with negative outcomes, including having passed away, were included.

*“If it is going to benefit other people and if you had passed away then it’s still going to be on the positive side isn’t it.” [Participant 01]*

#### Intent

For participants to be happy and comfortable with the secondary use of their information, it was clear that the intent of the use from the outset needed to be to benefit or help others.

*“It’s the intent, the how, the why they need to do that.” [Participant 12]*

In relation to COVID-19, participants understood that decisions around the release of health information had to be made quickly and on an unprecedented scale. Although they were quick to acknowledge that they felt there had been errors (e.g., cases where too much information, or potentially identifiable information, was made public), they were forgiving of this as the intent had been good and for the protection of others.

*“Bearing in mind; this is very new to everyone – the pandemic, and trying to manage it in the 20th Century. So, it’s purely the intent; why that information needs to be used?” [Participant 12]*

*“Well, I think in a pandemic it’s important. People want to know, have they been anywhere this person has gone – have they been anywhere near where this person has been – are they at high risk, or low risk? ... So, yeah I think in a pandemic, it was in everybody’s interest to know what is going on. Nobody wants the wool pulled over their eyes in situations like this.” [Participant 03]*

Regarding commercial or financial gain from the use of health information, participants were clear that the intent would no longer be to help others, rather, it would be for financial profit, and therefore their comfort with the use of their health information reduced.

*“Well then they are only doing it to make money. Not to help people.” [Participant 02]*

#### Communication

Participants described feelings of satisfaction and happiness that their health information could result in benefits to others. Although the idea that they were helping others just by having their health information used beyond their immediate care was something that made them feel good, participants frequently commented that they were not told if this was the case. It was clear that there needed to be improved communication around the use of health information for secondary purposes, including when its use had contributed to the greater good.

*“It’s about closing the feedback loop.” [Participant 10]*

*“That would be amazing [to be told when my health information had contributed to helping others]. That’s what the health section of the newspaper used to do.” [Participant 11]*

### Theme 2: sharing is important

Participants described an expectation that sharing of de-identified data for secondary purposes happened, that it was necessary and important. Secondary use of data was seen as needed to improve services and advance science, including for developing new treatments and technologies.

*“It’s all about bettering the system isn’t it?” [Participant 01]*

#### Benefits of sharing

Beyond just helping others, participants described that there were other benefits to the secondary use of health information. For example, with the AI/ML scenario, they stated benefits to patients receiving better or more timely care, as well as secondary benefits such as more efficient and timely services and the freeing up of clinicians for other needs.

*“Yeah, it’s that process that enables clinician time to be spent on other things and more money to be spent on treatment, then that’s a really positive thing.” [Participant 11]*

Although the potential for immediate and secondary benefits was a key reason for why secondary use was important, participants discussed that these benefits should not be outweighed by secondary harms. For example, if the AI resulted in more efficient diagnosis of cancer, without capacity for individuals to be treated in timely manner, it could result in more harm than benefit.

*“I think I would agree with it if it enabled increased diagnosis – as long as there was capacity to provide the treatment.” [Participant 11]*

#### Sharing with the patient

Although there was an expectation that sharing was happening, participants felt that if their information was being shared for these purposes, then it should also be shared with the patient themselves. Patients wanted easy access to their health information, which should be timely and not delayed.

*“The other thing that I would say about the current system is that I should be getting a copy of it. I think that’s something that isn’t done.” [Participant 10]*

*”I’ve asked for my records every time, and never got them, until now, and not straight away after I get out – probably around three weeks to a month after I get out. So, I really disagree with that…” [Participant 10]*

#### Harms of not sharing

Participants felt that sharing should happen as not sharing could be harmful and contribute to increased stigma and biases. This was particularly clear in relation to sharing for public health measures where not sharing of information might lead to the public seeking the information themselves though non credible sources. Further, although sensitive information (e.g., mental health information) needed to be treated carefully not sharing sensitive information for valid purposes could further reinforce existing stigma faced by patients in those services.

*“No. If the government health services don’t share it, people will go digging on Facebook to try and find [out], and then there’ll be impact – then you’ll get vigilante lynch mobs helping. It provides a level of protection to the person involved, and their family involved.” [Participant 11]*

### Theme 3: trust

It was evident that participants wanted to be able to trust the health service to respect, protect and use their data responsibly and not share it inappropriately.

*“Because maybe I’m a trusting person, I just believe the only reason they are wanting it is to try and help and benefit people for the future. The only way you can progress forward, using all relevant information at the time.” [Participant 01]*

*“I mean if they were handing it on to businesses to try and encourage me, those businesses were to contact me to try and encourage me to go and be with them so that they can make money out of me then I would have something to say.” [Participant 03]*

For example, participants described a lot of trust regarding health information being used for ML/AI due to not fully understanding these things (i.e., the technical side). They described that they often didn’t understand the details of how their data was being used in ML/AI developments, but this didn’t mean that they would not support it or that their health information couldn’t be used in this way. They wanted to trust that the health service would use their health information responsibly and ensure no harm was caused, regardless of whether they fully understood what was happening.

*“I mean, this is very much a matter that we would take on trust... So, when I say that it has my tacit approval, then that’s very much on that basis, with those provisos.” [Participant 07]*

Trust was described as something that was earned but would be difficult to repair once damaged.

*“It has to be earned, and obviously if there’s a breach of trust, then that is something that is very difficult to turn around.” [Participant 07]*

#### Governance

There was a need for good governance and oversight over the access and use of health information for participants to trust the health service to care for and protect their health information. Good governance included transparent processes and approvals for its use, along with ongoing auditing and monitoring. Participants discussed the importance of independent governance, ensuring that this was not just members of the health service management.

*“Well, that it should be subject to the full quality control over all aspects of what you’re suggesting, and that there should be an independent, probably third-party audit and approval of the process, and that basically is a stamp of approval for people like me, to be able to rely on…” [Participant 07]*

*“We need some fierce watchdogs. I just expect the panel to include some really robust, fierce watchdogs who are not necessarily let’s say the people that are on the [health service] management. So, it would be a matter of getting some fierce watchdogs whose credentials are suitable.” [Participant 07]*

The importance of local (i.e., NZ) oversight and governance was essential. This was seen as important to ensure data was handled respectfully, not misinterpreted, and outcomes of its use caused no harm. This was relevant to the research scenario where they saw benefit to international research being undertaken with NZ data but there were concerns that researchers overseas may not have the necessary understanding of the NZ context, in particular Māori (indigenous population of NZ) culture, to ensure this was used and interpreted correctly.

*“I’d trust the people who were in the [NZ] team to be able to allow for any kind of specific things about New Zealand… between Australia and New Zealand, probably not that many differences, but between New Zealand and say the United States, there’d be a lot of differences. It’s not for [international researchers] to decide what they are, but I’d just leave it to the [NZ] team to be able to deal with those aspects of it.” [Participant 10]*

Onward sharing or further use of the data worried participants. Having New Zealanders involved was seen as a way to prevent this from happening.

*“I think some New Zealanders should still be involved. I think they should be to a certain extent, and also what happens after that information is used – will that be continuous – will it be forever? If Australia is asked to use that information with another country past Australia – to share that information, what happens in that scenario?” [Participant 08]*

Participants commented that although there needed to be a degree of independent governance and audit, the responsibility for the safe use of health information belonged to the health service. In this way the responsibility for preventing harm from the sharing or use of health information was seen to lie with the original health service that collected the data. Therefore, the original health service needed to remain involved in ongoing use of the data.

Further, alongside good governance, participants emphasised the importance of clinician oversight over both the use of data and the output of the data. For example, although they saw a clear benefit in a future involving AI, it was essential to see their clinician’s oversight and support of the AI in practice to be comfortable with it. Further, participants did not want clinicians removed from clinical care or the patient’s choice to see a human doctor face-to-face taken away.

#### Privacy and de-identification

A key element to trust was the protection of individual privacy and ensuring de-identification was done correctly. It was clear that participants wanted their identifiable information protected and access to this audited. If they could trust that this was happening, they would be comfortable with the use of their health information for secondary purposes across the scenarios.

*“Well, once again, I think it’s okay as long as your name and all your details are taken off. So, your privacy remains your privacy. I can’t see the harm in using your records, as long as they don’t know who you are.” [Participant 03]*

If the patient’s privacy could not be protected or the data adequately de-identified, then explicit consent for its use would be needed. This covered not just identifiable information such as their contact information but also sensitive information where an individual or community could be stigmatised or harmed.

When presented with the possibility of re-identifying a person from the data, such as researchers re-identifying patients from a registry to offer them new treatments or research studies, participants wanted this to be done by their original health service (i.e., not done by researchers or clinicians at another health service conducting the research or offering the new treatment).

*“Yeah, so I don’t think that’s appropriate. I think it would need to be your initial team that you gave permission to store the data, that actually is in contact with you, even if that is forwarding an email from a different researcher… I think it’s a good idea, but it’s where it comes from, and out of the blue is never a good way, as opposed to the original links, to who you originally gave permission to. I wouldn’t expect it from an individual clinician; I’d expect it from the service.” [Participant 11]*

The importance of privacy as a fundamental element to trust was discussed in relation to the release of health information to the public for public health purposes. Not only was it paramount that the public release of health information excluded personal identifiable information (e.g., name, address), but that this also excluded information that identified groups or communities (e.g., churches).

*“I have no issue with it being shared with health officials. As you stated; they need to follow-up with the patient, and do the contact-tracing and all that sort of stuff. So, that’s fine. The issue becomes; what do you share with the media and other private organisations who may not be directly involved in the person’s health care or follow-up. I don’t think things like names, race, or anything like that should be shared with the media. It should literally be; a person in area – visited these locations at these dates and times. That’s really all the media and the public need to know at large. Yeah, you don’t need the media to go off and say; a Māori blah-blah-blah. Yeah, if it was a non-Māori – or non-PI [Pacific Island], they’ll just say; a person in [suburb name] contracted Covid. So, it takes away that power of the press to vilify any particular race. It’s even irrelevant whether they’re a church-goer or anything like that.” [Participant 06]*

#### Security and storage

Assurances that information was stored securely was also needed for there to be trust. When asked about any concerns they had with the current use of their health information, participants described concerns around security, potential hacking, and unauthorised access.

*“The only concern, as I said before, is the hacking problem. That’s the only concern I have.” [Participant 09]*

*“I think, yes - I think as long as the information is secure. I’m thinking of what happened at [health service name]. I don’t know what implications that had about information being sent out. I don’t understand that, but I just feel that the [health service] have to really tighten up their processes.” [Participant 05]*

Digital storage of health information was perceived as allowing for better protection and security of health information. In contrast, participants described how this was not case with health information shared verbally which often gave no protection to the individual’s privacy.

*“So, one of my concerns is… when you go into the hospital system, everyone’s saying, from the ward clerks to ED – what’s your name – you’re shouting that out – what’s your problem – the nurse comes out to you in the waiting room – everyone can hear your business. So, from a confidentiality perspective – even when the doctors are doing their rounds, and you’re in a shared room, everyone can hear what your problem is. So, that’s where I think there’s a massive issue in the system. We talk about confidentiality, but you’re already having to say what your name is, what your problem is. In ED you have to actually shout now that they’ve got screens up during Covid. So, there’s no more privacy. …it’s when you’re having to sort of shout out your information, and doctors are talking to you about your problem, and everyone else in the room can hear you.” [Participant 12]*

#### Transparency and communication

A final factor for participants to trust their health service was the need for transparency and communication around the use of their health information.

*“Communication is the key. Have good communication. Have a good rapport with your patients.” [Participant 12]*

As described above, it was clear that there needed to be improved communication around the use of health information for secondary purposes. Communication should include the purpose and intent for use, and the outcomes. It was also highlighted that consent forms should be regularly updated to include potential future uses as these arose.

### Theme 4: respect

Participants wanted to be respected and their health information to be respected. This included respect for the data and its value, respect for the person behind the data and their privacy, and respect for the consent process.

*“If you’re in a middle of an episode and you’re saying, look – I don’t consent to these people having information about you, then it’s important that be respected.” [Participant 10]*

#### All information is valuable

Participants clearly understood the value of health information and how profits could be made from its use. Respecting the value of health information meant that it needed to be protected, not released outside the health service for anyone to do what they wanted with. If profit was to be made by a company, it was clear that many participants expected that this would be passed onto the health service or them individually (or their whānau (family) after their passing).

*“I feel they should come up with a remuneration system that actually – I should get paid if my data is getting used; I think that’s how it should – yeah. I think that the individual should be paid. I think the [health service] should be paid, as well as there should be a cut for an individual involved, because I think, firstly the person who’s given the data – the data is being used, and should be paid for that, but I also think that the collation of the information and storage basically takes time, and they should get paid for it, too… Yes, and I think it should be substantial. If you’re going to use it – I think their margins are massive, so there’s definitely space to.” [Participant 08]*

Participants highlighted the importance of all health information being treated as valuable with no type of information being more valuable than other types. Although the importance of treating sensitive (e.g., mental health, sexual health) information carefully was discussed, not using this information was seen as disrespectful and contributing to biases and stigma. The secondary use of health information was perceived as key to advances in healthcare, and so if this was not done across all areas of health, including sensitive areas, those areas would be left behind.

*“I mean, even [mental health services], examples which I think are really important – that information can be used, is when it comes to seclusion and restraint. Those figures are really important… I would want those statistics to be counted… [they can’t exclude some information] yeah, especially if you’re trying to move in the right direction, in terms of ensuring that people get better care.” [Participant 10]*

#### Sharing with the patient

It was clear that patients needed to have access to their health information to feel respected. Regardless of the type of information, if the health system could share it, it should be shared with the patient first.

*“It’s your information; I think people should be able to access it.” [Participant 05]*

*“We have a saying - people with lived experience; nothing about me, without me. I think that is just vitally important.” [Participant 10]*

Again, it was highlighted that all patients and types of health information should be respected equally; therefore, access to health information needed to be for everyone regardless of the type of health information (sensitive or not).

*“If you had a mammogram result and you had been diagnosed with breast cancer, you probably don’t want anybody else to know about that before you do [and] I don’t think that probably happens, eh? Well, yeah you don’t have as many rights in mental health as you do [in other areas]. I mean, they don’t respect you – you don’t have the same rights [to your information].” [Participant 10]*

Beyond just having access, patients also wanted to be able to contribute to the health information the health service had about them. They wanted to have the option to contribute, add or edit their information. Participants described concerns around the accuracy of health information and that there were cases of incorrect information being used repeatedly. They reported a lack of clear processes for having this corrected or even the opportunity to discuss the errors. Therefore, mechanisms to identify and edit/have their information reviewed were vital.

*“I feel there’s a lot of cut and paste going on. If they get one thing wrong, it continues.” [Participant 05]*

#### Security and storage

Ensuring data was adequately protected was a key part of respecting it. Adequate protection involved it being stored locally. Participants wanted their data to remain in the NZ health system and not sent outside where there was risk it could be on-shared or disrespected.

*“I think that they shouldn’t be able to migrate that data over to – I think firstly, if they’re going to store data, it should be stored locally, in New Zealand; I don’t think they should be taking that data and storing it overseas. I think that information is New Zealand’s.” [Participant 08]*

Sharing health information outside the health system was seen as removing it from its context which is vital for it to be used and interpreted respectfully. Participants highlighted that without the context, the complete picture would not be considered, including socio-cultural factors.

*“For a NZ or Kiwi-based researcher interpreting the data, they may have to take into account the different cultures and how New Zealanders behave, because we won’t necessarily behave in the same way as an Australian or whatever, in terms of trying to figure out what to do based on that data.” [Participant 06]*

#### Transparency and communication

Similar to trust, transparency and communication were also crucial to respect. Being transparent about what information was collected, how it was stored, and how it might be used (proactively) was seen as important.

*“I have no issue with what they want to do; it’s just nice to know that they’re doing it… It’s about the transparency. So, why are they needing the information – what they’re going to do with that information, and what the outcome of that information is. So, once again, it comes back to that transparency.” [Participant 12]*

*“I think they need to have a conversation… You have got to have an understanding of why, and how that information will be used, and what the outcome is – what’s the purpose – and what’s the outcome.” [Participant 12]*

*“I think there’s a real obligation for the clinician to be transparent around what they can see, as well. I think when you go and see somebody like the dentist, the dental clinic, or the doctor, it’s quite clear that they’re taking notes and that they’re recording; I guess probably what’s not clear is, oh you’ve had a heart attack – you’re now on the register.” [Participant 11]*

Not communicating details about health information and its use, including technical details, was a sign of disrespect. Even if a patient would not understand it, knowing that details were available was important.

*“Some people wouldn’t, but I would read it [security details], it would be double Dutch, and it would be over my head.” [Participant 03]*

## Discussion

This study has identified four interrelated overarching themes representing the main issues for participants around secondary use of their personal health information: (1) That health information should be used to help and benefit others, (2) that sharing of de-identified data for secondary purposes is necessary and important, (3) that personal health information is valuable and should be respected, and (4) that people want to be able to trust the health service to respect, protect and use their data responsibly and not share it inappropriately.

Although the findings from this study are similar to those from previous studies,[5, 7] they are an important addition to the literature in this field due to the NZ context, the focus on patients when they are encountering the health service, and the study was conducted after the start of the COVID-19 pandemic which for many gave a new understanding of the potential for the use and sharing of health information. As previous studies, and our earlier surveys, have highlighted, the support for the secondary use of health information is conditional.[5–7, 15] Key conditions for patient support of secondary use of health information include the use being for the greater good and that they can trust their health service (or those who collected the data) to protect and care for their health information. Not only is it paramount that the secondary use of health information is for the direct purpose of benefiting others, but there also needs to be assurances that its use will not result in immediate or secondary harm. The importance of trust is well established as being essential to public and patient perceptions of the use of their health information for research,[5] and this study highlighted its importance is not just limited to research purposes. Health services must actively work to build and maintain trust and minimise opportunities for mistrust to arise.

Reinforcing findings from previous studies, concerns around privacy are at the forefront of people’s minds when discussing the secondary use of their health information.[5, 7] Assurances that their information will be correctly de-identified, that it is stored securely, and that there will be good governance over this, are vital for patients to feel comfortable. The importance of privacy extends beyond the individual to respect the privacy of the individual’s community. This was apparent when discussing the sharing of health information for COVID-19 surveillance. Patients felt that sharing of individual health information should not only respect the privacy of the individual case (e.g., their address should not be released) but also the privacy of their community (e.g., their cultural group, church). Although the de-identification of health information is complicated, this study highlighted that people need to be able to trust that their health service would be doing this to the best of their ability.

Participants in the current study were well aware of the ethical conundrum around the secondary use of health information.[23–27] Although there was a clear desire for autonomy over their health information, they recognised that this did not necessarily mean that they would be able to consent to every use. Their desire to contribute to improving the lives of others, alongside the awareness that it was in some cases impossible or unethical to remove an individual’s data from a dataset, meant that they were understanding of secondary use of their de-identified data in the absence of explicit individual consent. Participants were aware that if individual consent was required, there would likely be biases.[28, 29]. Although they acknowledged that the health service didn’t need to contact them individually regarding the use of their de-identified data, they did expect good governance and approvals over its use, and open communication and transparency from the health service about how it was using health information, potential future uses, and the outcomes of past use. This is not new and the need for transparency and improved communication around the use of health information has been well documented in the literature for years,[5, 13, 30] although the lack of awareness of participants in this study of the use of their health information indicates that progress in this space has been limited. Discussions around how communication around health information use should occur found that this needed to be multifaceted to ensure all members of the community had access to the information if, and when, they needed it, noting that often the need for information arises because of a particular event (e.g., cybersecurity threat, diagnosis) rather than wanting regular routine information disseminated to them.

### Strengths and limitations

Although data sufficiency was achieved, the small sample size limits the generalisability of the findings. Further, the sample, although diverse, did not have representation of some health services e.g., gynaecology. Previous research has reported varying comfort across different information types.[31] There was also a lack of Māori and Pacific Island participants limiting the generalisability of the findings to these key population groups. The study was initially designed to be conducted face-to-face within the health system to capture people’s views while physically engaging with the system. COVID-19 restrictions meant that this, and in-person recruitment, was not possible. This may have impacted the results by limiting the ability to build rapport and by the increased cognitive load associated with video conferencing. Although the generalisability of the findings is limited by the small sample size and representation of the sample, it builds on the broader survey work to provide a greater understanding of some of the quantitative responses.

#### Implications

From this work the following key considerations were identified for the use of patient health information beyond the delivery of individual health care:

1. Health information should be respected and only used for good. Therefore, secondary use of health information should not contribute to harm or biases.
2. Secondary use of health information is expected and necessary for ongoing improvements in health care, but this should only occur where the intent is to benefit the population.
3. Individual privacy and confidentiality need to be prioritised as well as the privacy of the communities the individual is a member of. Ensuring de-identification is done correctly should be prioritised.
4. There should be clear communication and transparency around the use and protection of personal health information. Transparency and adequate communication are needed for the health system to be trusted to care for and protect individual health information. Communication should be proactive and multi-levelled, with lay summaries available first but technical information (e.g., security processes) also available.
5. There should be clear governance of health information, including clear processes and approvals for its use, with auditing and monitoring. The responsibility for preventing harm from the sharing of health information is considered to lie with the original health service, and they should remain involved in the ongoing use of the data to ensure no harm or biases result from its use.
6. Health information must be stored securely and protected from data breaches and hacking.
7. Health information should remain in the health system. It should not be shared outside the health sector or for commercial uses or financial gain. This includes sharing with research institutions (e.g., Universities) and non-governmental organisations. If the information is to be shared outside the health system then individual consent should be obtained first. Ways for external researchers and organisations to have secure access to health information within the health system should be explored. With advances in secure cloud environments, there are opportunities for health services to retain guardianship and protection of their patient data while allowing approved researchers and analysts auditable access for secondary uses.
8. Patients should be given access to their health information and the ability to update or add to their information. Not only are there clear benefits to patients having access to their information, but this ensures patients will have a better understanding of the implications of their consent to its use beyond their care.

## Conclusion

Current users of health services are supportive of their health information being used to help others, advance science, and contribute to the greater good but their support is conditional. People need to be able to trust the health service to protect, care for, and respect their health information and ensure no harm comes from its use. This study has identified key considerations for services, researchers, and clinicians to reflect on when using patient health information for secondary purposes to ensure they use it in a patient-informed way.

## Electronic supplementary material

Below is the link to the electronic supplementary material.

Supplementary Material 1 COREQ

Supplementary Material 2 Interview guide: Waitematā DHB patient interviews on data use

## Data Availability

The research team will consider reasonable requests for sharing of de-identified data. Requests should be made to the corresponding author.

## Abbreviations

AI: Artificial Intelligence
ML: Machine Learning
NZ: New Zealand
