# Supplementary material for: Exploring patient perspectives on the secondary use of their personal health information: an interview study
Source: BMC Med Inform Decis Mak. 2023 Apr 11;23:66. doi: 10.1186/s12911-023-02143-1 (PMC10088161; doi:10.1186/s12911-023-02143-1)
Supplement: Supplementary file 2 — Supplementary Material 2 Interview guide: Waitematā DHB patient interviews on data use [file 12911_2023_2143_MOESM2_ESM.pdf]

Supplementary File 1 for manuscript: Dobson, R. Wihongi, H. Whittaker, R. Exploring patient perspectives on the secondary use of their personal health information: An interview study.

<u>Interview guide: Waitematā DHB patient interviews on data use</u>

Section 1. Current use of health information

Health information is any information that the District Health Board (DHB) collects on you and your health. This includes information from every visit and contact you have had with a service at the DHB. This might include information about any visits to ED (e.g., for an accident), information about surgeries and treatments you have had at the hospital, as well as any visit to community services and clinics (e.g., diabetes clinic, dental services, mental health). It also includes what we call demographic information such as your legal name, contact details, date of birth, and ethnicity. It isn't just written information it also includes things like x-rays and images that you may have had taken. This information is used by your clinicians (e.g., doctors, nurses) to care for you.

Your clinician will use information from your current visit as well as previous visits to our services to make decisions about your health care. The first time you used a DHB service, you will have consented to the DHB collecting this information (or your parents consented if you were a child). Currently, the DHB uses your health information in the following ways:

- Your doctor and other clinicians use it to guide your current treatment and care. They share the information with other people who look after your healthcare such as your GP or clinicians at another hospital.
- Your de-identified information is used for statistics which are used to monitor the DHB services to ensure that they are running smoothly

For your health information to be used in other ways, such as for research, typically you will have to provide consent for this, and if you can't be contacted, they won't be able to use your health information.

Q1: What do you think about how the DHB currently uses your health information?

Prompts: Is it what you thought happened? Yes/No. If not, how does it differ? Are there any parts that you are uncomfortable with? Do you have any concerns?

Section 2. Possible uses of health information

Next, I want to discuss with you some scenarios of how patient health information could be used. There are 5 different ways that I will describe.

<u>Scenario 1:</u> National and international research is developing ways for computers to read some radiology and other images (e.g., chest x-rays or CT scans, eye screens, mammograms) with greater accuracy than human clinicians. This involves a computer programme interpreting a person's x-ray based on data from thousands of patients who have had the same type of x-ray in the past. The more people's data (e.g., scans) that are used the more accurate the computer programme will be. To develop the computer programme the DHB could use all the mammogram images and results of these (e.g., breast cancer or no cancer) from every patient who has had a mammogram at the DHB. This could mean that a computer can read mammograms of people who have mammograms in the future at the DHB making the diagnosis of breast cancer more accurate, quicker, and cheaper than traditional methods.

Q2.1: What do you think about your health information being used in this way?

Prompts: What do you think about your whānau health information being used in this way? [including after they have passed away]

To assess how accurate the system is they may want to link the scans with future health information. For example, did the person get diagnosed with cancer in the future. What do you think about your health information being used in this way?

If this happened, the companies that own the screening machines and computer systems would have access to the health information (e.g., scans/mammograms) and may use it to create their own computer programmes to interpret scans in other places (e.g., other countries). Does this change how you feel about your health information being used in this way? If yes, how does it change?

If this happened the company could make money off selling the computer programme. This would mean they are profiting off something they developed using the health information of yourself and other patients. How do you feel about this?

Scenario 2: In New Zealand there are a number of registries. Registries are databases of information for all the people that have a certain condition or type of treatment. For example, if you have a heart attack your information could be added to the cardiac register. Your health information is de-identified when it is added to the registry. Clinicians and researchers can then look at trends and outcomes for people in the registry to learn things like the types of people having heart attacks or the outcomes of certain treatments in certain types of people.

Q2.2: What do you think about your health information being used in this way?

Prompts: What do you think about your whānau health information being used in this way? [including after they have passed away] These registries can be very useful for identifying people who might benefit from a new treatment/service or who could be eligible for a research study. How would you feel about someone not from your medical/clinical team (e.g., a researcher from a university or a doctor you may not have met from another NZ hospital) contacting you because they have seen from the registry that you could benefit from a new treatment/service/research?

Scenario 3: Sometimes to determine whether someone should have a particular treatment a calculator is used. Certain information about the condition is put into the calculator to produce a score which can then be used to determine whether they meet the required threshold to undergo the treatment. For the calculator to work the health information of lots of patients who have previously had the condition and treatment is needed. Often the calculators that exist use data from overseas. It would be more relevant and accurate to use health information from New Zealanders to inform these calculators but to do this, they would need to be able to access the health information of all the people who had had the condition at this DHB even if they had passed away. The health information would be de-identified, but these people may not have consented to their information being used in this way.

Q2.3: What do you think about your health information being used in this way?

Prompts: What do you think about your whānau health information being used in this way? [including after they have passed away]

Scenario 4: Clinicians are interested in looking at how well their service is doing at helping people to recover faster from hip replacement surgery. To do this, they want to access the health information of everyone who underwent that surgery in their hospital in a particular year. This would be de-

identified data but include details such as demographics, existing health conditions/diseases, specific test results, medications, allergies, and surgery outcomes. They may then try improving the service (e.g., changing the physiotherapy given post-surgery) and monitor any changes in the data to see whether it has been successful. As part of this, the clinicians may find it useful to compare the data from their hospital with other hospitals doing hip replacement surgeries. To do this, they might need to work with clinicians from other hospitals or researchers from partner institutions (e.g., universities) to do the analysis. This would involve sharing the de-identified data with these clinicians/researchers. This sharing of health information would be for the benefit of others rather than yourself personally.

Q2.4: What do you think about your health information being used in this way?

Prompts: What do you think about your whānau health information being used in this way? [including after they have passed away] Does how you feel about this use of your health information change if it involves sharing of health information with institutions outside NZ (e.g., Australia)?

Scenario 5: When people have an infectious disease like COVID-19 certain information is made public so that actions can be taken to identify and prevent the further spread of the condition to others. This type of information was commonly included in the briefings by Director-General of Health during the COVID-19 pandemic. The DHB will share information about the person with COVID-19 (including identifiable information) with other organisations within the health system (e.g., the Ministry of Health or the Regional Public Health Service) who will then also make some of the de-identified information available to the public and media (e.g., genetic variant, infectious period, places they visited while infectious, where they may have caught the virus).

Q2.5: What do you think about your health information being used in this way?

Prompts: What do you think about your whānau health information being used in this way? [including after they have passed away]

The next few questions are just about the use of your health information generally.

Section 3: Consent to use health information

Q3: Are there any situations [types of information] where you want the health service to get your consent [permission] to use your health information?

Prompts: How would you want to be asked for consent? How often do you want to be reminded about consent (given the option to review your consent)?

Section 4: Access to personal health information

Q4: How do you think people should be able to access their own health information?

Section 5: Communication of use of personal health information

Q5: How do you think the health service should communicate about how they use people's health information?

Prompts: When and where should communication occur? What should the health service include in the communication?
